# Extra uterine development of preterm kidneys

**DOI:** 10.1007/s00467-018-3899-1

**Published:** 2018-03-02

**Authors:** Yogavijayan Kandasamy, Donna Rudd, Roger Smith, Eugenie R Lumbers, Ian MR Wright

**Affiliations:** 1 Department of Neonatology, The Townsville Hospital, 100 Angus Smith Drive, Douglas, QLD 4814 Australia; 2 Mothers and Babies Research Centre, Hunter Medical Research Institute, The University of Newcastle, Callaghan, NSW 2308 Australia; 3 College of Public Health, Medical and Veterinary Sciences, The James Cook University, Douglas, QLD 4814 Australia; 4 School of Biomedical Sciences and Pharmacy, University of Newcastle, Callaghan, NSW 2308 Australia; 5 Illawarra Health and Medical Research Institute and Graduate Medicine, Faculty of Science, Medicine and Health, University of Wollongong, Wollongong, NSW 2522 Australia

**Keywords:** Premature, Preterm, Renal volume, Estimated glomeruli filtration rate

## Abstract

**Objective:**

We carried out a study to determine the impact of prematurity on renal development. The primary outcomes measured were nephrinuria and albuminuria; renal volume and glomerular filtration rate were the secondary outcomes.

**Methods:**

Preterm neonates born at less than 28 weeks of gestation, with birth weight between 10th and 90th centile (appropriate for gestational age), were recruited and underwent assessments at 28, 32 and 37 weeks postmenstrual age (PMA).

**Results:**

Fifty-three premature neonates and 31 term neonates (control) were recruited. The median gestational age of the premature neonates was 26.4 [24.7–27.4] weeks, with a mean birth weight of 886 (179) g. The mean gestational age of term neonates was 39.1 (1.2) weeks and the mean birth weight was 3406 (406) g. The median age of the term neonates was 6.5 [3.0–12.5] days. The total kidney volume (TKV) almost doubled from 10.3 (2.9) cm^3^ at 28 weeks PMA to 19.2 (3.7) cm^3^ at 37 weeks PMA (*P* = 0.0001). TKV at 37 weeks PMA was significantly smaller compared to term neonates (19.2 (3.7) vs 26.3 (7.0) cm^3^; *P* = 0.0001). However, there was no significant difference in estimated glomerular filtration rate (eGFR) between premature neonates (at 37 weeks PMA) and term neonates (control) (43.5 [39.7–48.9] vs. 42.0 [38.2–50.0] mL/min/1.73 m^2^; *P* = 0.75). There was a statistically significant decline in nephrin-creatinine ratio and albumin-creatinine ratio from 32 to 37 weeks PMA.

**Conclusions:**

Despite having a smaller renal volume (and fewer nephrons), extremely premature neonates achieve similar eGFRs at corrected term as term-born neonates, likely through single nephron hyperfiltration. Extremely premature neonates also show evidence of glomerular injury.

## Introduction

There are more than 15 million preterm (birth before 37 completed weeks of gestation) births globally per year, and the number is increasing [1]. Advances in perinatal care and the use of antenatal glucocorticoids, antibiotics, surfactant, and improved ventilation strategies have contributed to improving outcomes for premature infants. Improved health care in developing countries has resulted in an increased number of premature neonates surviving to adulthood [2]. Prematurity is associated with long-term morbidities such as cognitive, psychological, neurological and visual deficits [3]. In recent years, there is increasing evidence to indicate that prematurity is an independent risk factor for chronic renal diseases (CKD) [4, 5]. The pathogenesis of CKD in premature neonates remains to be fully understood. The currently available evidence indicates that prematurity results in reduced nephron endowment by interrupting normal organogenesis [5]. Nephron number can be determined by histopathological examination [6]. Renal volume measurements have also been shown to have good correlation with nephron number [7, 8].

Currently, microalbuminuria is used as an early indication of glomerular pathology [9]. Measuring urine albumin over a 24-h period is often not clinically practical, particularly in neonates; hence, urine spot tests and measurement of urine albumin-creatinine ratio (ACR) have become popular in clinical practice. Nephrin is a 180-kD transmembrane protein expressed in glomerular podocytes. It forms an integral part of podocytes, which, together with endothelial cells and the basement membrane, form the glomerular filtration barrier. Podocytopathies lead to the detectable levels of nephrin in the urine. Both animal and human studies have demonstrated that nephrinuria occurs early in glomerular injury, often preceding albuminuria, and that there is a positive correlation between nephrinuria and the severity of renal diseases [10]. Urinary nephrin analysis thus has the potential to be an important biomarker of early glomerular injury [10].

We carried out a study to determine the impact of prematurity on renal development in a cohort of extremely premature neonates. The primary outcomes were nephrinuria and albuminuria; renal volume (a surrogate for nephron number) and glomerular filtration rate were secondary outcomes.

## Materials and methods

### Study population

This prospective case-control study was conducted in the Department of Neonatology, Townsville Hospital, Queensland, Australia. The hospital is a tertiary perinatal centre that caters for a region with more than 10,000 births each year. The study was conducted from August 2014 until October 2016. The neonates recruited in this study are part of a larger ongoing study that investigates the relationship between prematurity, retinal vascularisation and renal development in preterm and low birth weight neonates. Preterm neonates at less than 28 weeks of gestation (extremely premature neonates), with birth weights between the 10th and 90th centile (appropriate for gestational age (AGA)), admitted to the neonatal department during the study period were eligible to participate in this study. Neonates with congenital abnormalities or syndromes were excluded. Preterm neonates were recruited and followed until discharge. Once recruited, the preterm neonates underwent their first assessment at 28 weeks postmenstrual age (PMA) and a second assessment at 32 weeks PMA and a final evaluation was undertaken at 37 weeks PMA. PMA for the preterm neonates was defined as follows: PMA = gestational age at birth (weeks) + postnatal age in weeks. A cohort of term neonates admitted to the neonatal unit with minor neonatal conditions such as jaundice or feeding problems were recruited into the control group.

### Data collection

During each assessment, the neonates underwent renal ultrasonography and venepuncture for cystatin C (CysC) measurements with a concurrent random urine analysis for albumin-creatinine (ACR) and nephrin-creatinine (NCR) ratio measurements.

#### Urine analysis for albumin and nephrin

Analysis of microalbumin was performed on randomly collected urine samples using the Beckman Coulter microalbumin immunoturbidimetric assay on the automated Unicel DxC analyzer (Beckman Coulter, Australia). The assay was calibrated using the SYNCHRON Systems MA calibrator specifically for urine assays (Beckman Australia), and this calibrator is traceable to the International Federation of Clinical Chemistry reference.

Analysis of human nephrin was performed on randomly collected urine samples using the Exocell Human-Nephrin ELIZA assay (Exocell, Philadelphia, USA). The range of the assay is 0.031 to 2.0 μg/mL. The intra- and inter-assay coefficients of variation for samples with concentrations in this range have been found to be < 10%, and this was confirmed under our experimental conditions [11]. The Nephrin ELISA uses rat nephrin in a urine matrix as a standard, and a rabbit polyclonal antibody raised against the N-terminal portion of human nephrin that cross-reacts with nephrin of several mammalian species including human, rat and mouse.

#### Cystatin C measurement

Cystatin C (CysC) concentrations were measured in serum samples using an immunoturbidimetric assay designed for use on the Beckman AU480 automated analyzer platform (Gentian, Australia). The Gentian CysC calibrator was standardized against the international standard ERM-DA471. The range of the assay is 0.34 to 7.95 mg/L. The intrai and inter-assay precisions for samples with concentrations in this range have been found to be < 10% (Beckman), and this was confirmed under our experimental conditions. Estimated glomerular filtration rate (eGFR) was calculated using the Zappitelli CysC eGFR equation (*GFR (ml/min per 1.73 m*^*2*^*) = 75.94/[serum cystatin C*^*1.17*^*]*) [12].

#### Renal ultrasonography

All renal ultrasounds were obtained using the Philips IU22 Ultrasound System (Philips Healthcare, Andover, MA, USA) with a compact (small footprint) curved linear 5–8 MHz frequency transducer. All renal scans were performed by the same sonographer, who was blinded to the clinical information. Intra-class coefficient for intra-observer variability was 0.85 (95% confidence interval 0.73–0.91). Renal length (*L*), anteroposterior diameter (AP) and transverse diameter (*W*) were measured for both kidneys. Kidney volume (KV; cm^3^) was calculated according to the following formula: KV = (*π* ∕ 6 × *L* × *W* × AP) [13]. The total kidney volume (TKV) (right KV + left KV) was also calculated.

### Statistical analysis

The normality of the variables was determined by the D’Agostino-Pearson test [14]. The results are expressed as the means [standard deviation (SD)] for continuous, normally distributed data and as median [interquartile range (IQR)] for continuous, non-normally distributed data. Comparisons of means of normally distributed data were made using *t* tests, and Mann-Whitney tests were used for non-normally distributed data. *P* value < 0.05 was considered statistically significant. Statistical analyses were performed using MedCalc for Windows, version 16.4.3 (MedCalc Software, Ostend, Belgium).

## Results

During the study, 131 premature neonates less than 28 weeks gestation were admitted to the neonatal unit. There were nine deaths. Consent was obtained in 59 neonates, and one infant was excluded because of hydronephrosis. Complete data sets were available in 53 neonates. During the same period, 31 term neonates were recruited as the control group. The median gestational age of the premature cohort was 26.4 [24.7–27.4] weeks, with a mean birth weight of 886 (179) g. At 37 weeks PMA, the mean weight of the premature group was 2416 (328) g. The mean gestational age of term neonates was 39.1 (1.2) weeks and the mean birth weight was 3406 (406) g.

There was a statistically significant decline in NCR from 32 to 37 weeks PMA (0.07 [0.04–0.18] to 0.04 [0.03–0.09] g/mol; *P* = 0.028). However, there was no significant difference between NCR at 37 weeks PMA and levels in term neonates (0.04 [0.03–0.09] vs 0.05 [0.03–0.14] g/mol; *P* = 0.42). There was also a statistically significant in decline of ACR from 32 to 37 weeks PMA (24.5 [14.5–43.5] to 6.7 [4.2–23.8] g/mol; *P* = 0.0009). ACR at 37 weeks PMA was however significantly higher compared to controls (6.7 [4.2–23.8] vs 5.1 [1.9–6.8] g/mol; *P* = 0.022).

**Fig. 1:**
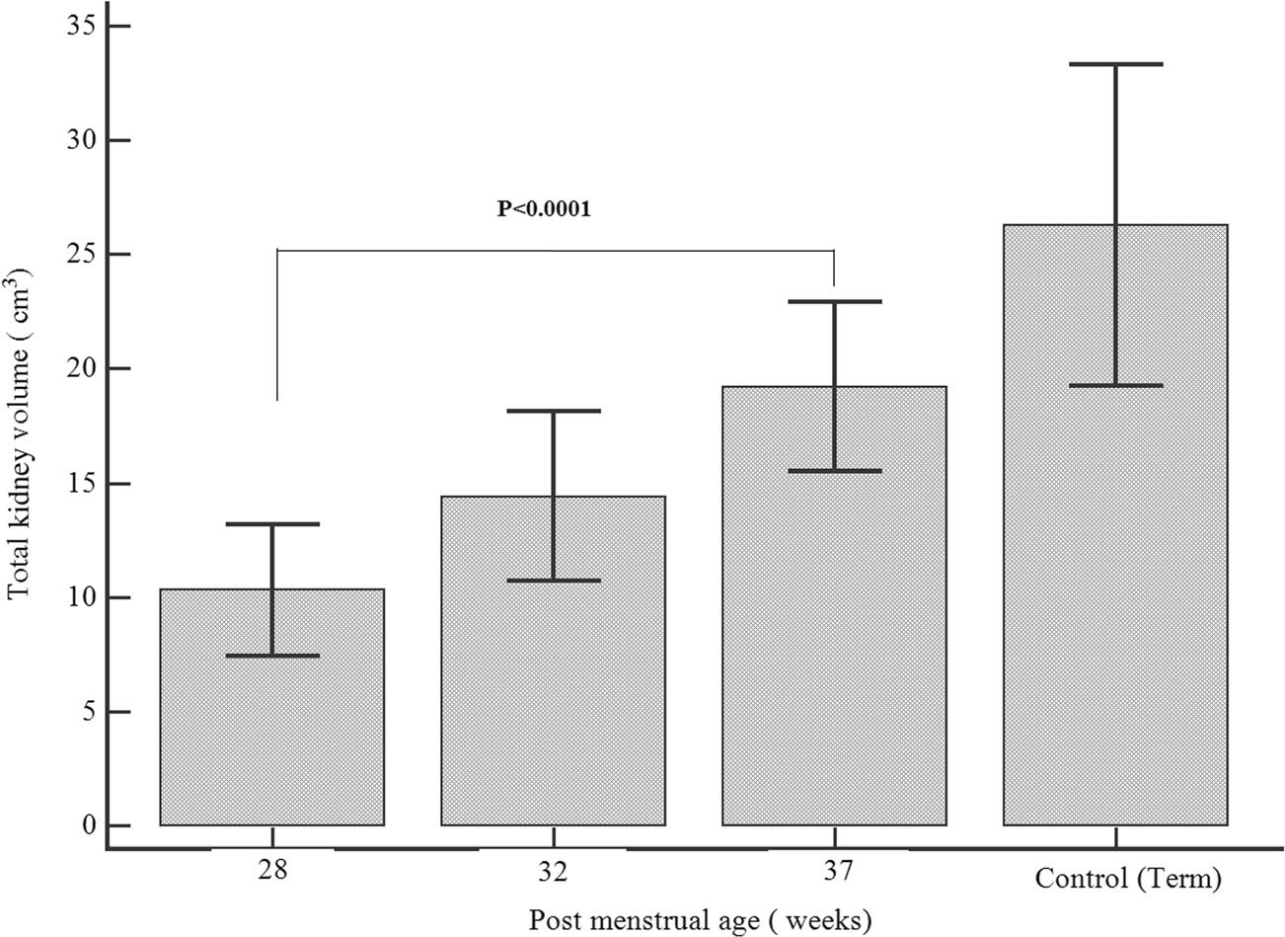
Total kidney volume [(TKV) mean standard deviation (SD)] in premature neonates at different postmenstrual ages with term neonates

**Fig. 2:**
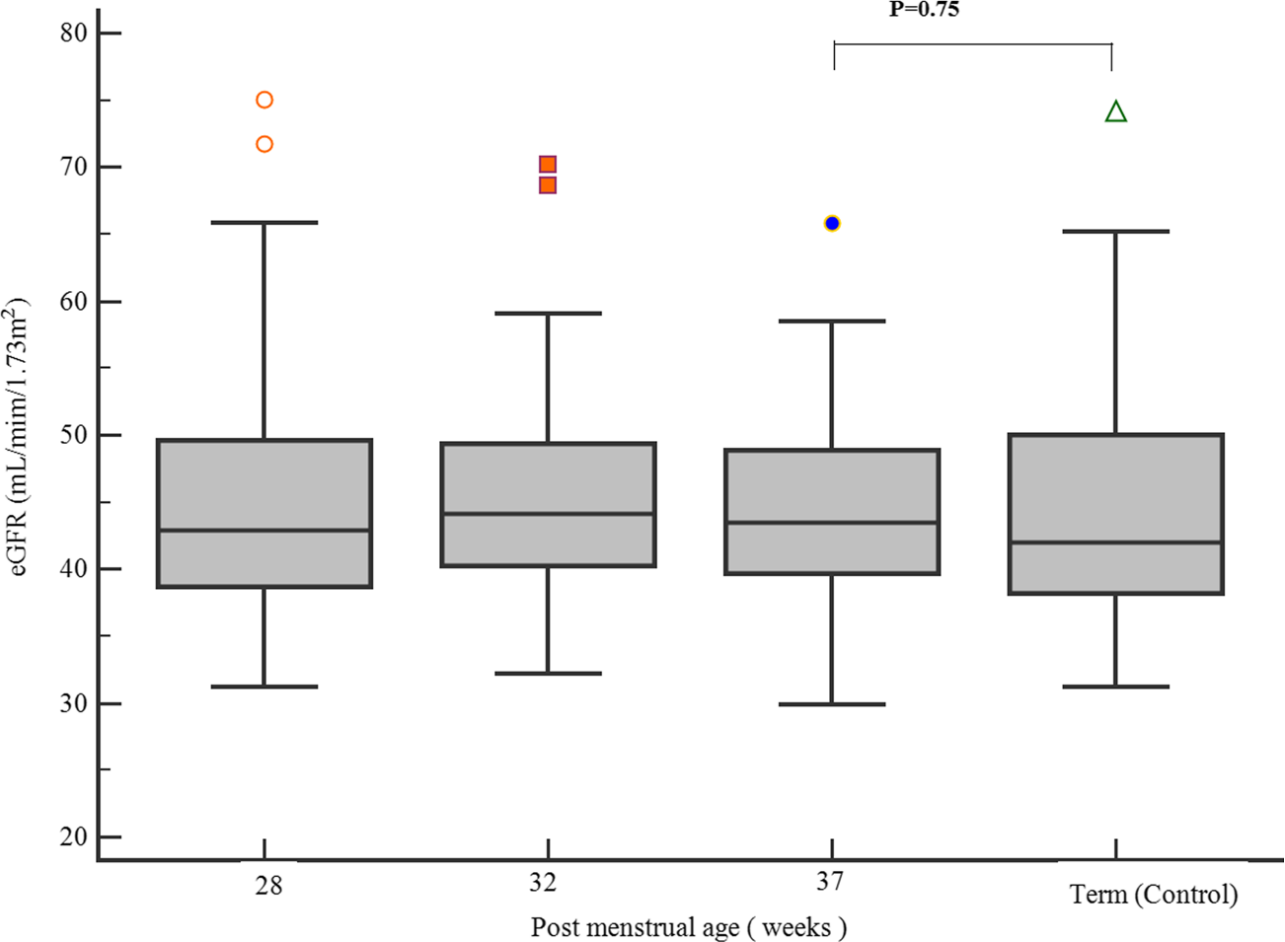
Comparison of estimated glomerular filtration rate (eGFR) in premature neonates at different postmenstrual ages with term neonates

The TKV almost doubled from 10.3 (2.9) cm^3^ at 28 weeks PMA to 19.2 (3.7) cm^3^ by 37 weeks PMA (*P* < 0.0001). TKV at 37 weeks PMA was significantly smaller compared to term control (19.2 (3.7) vs 26.3 (7.0) cm^3^; *P* < 0.0001). Figure 1 shows the changes in TKV in the early postnatal period. Figure 2 shows eGFR in the premature neonates at the different postmenstrual age in comparison to term. There was no significant change in the eGFR from 28 to 37 weeks PMA (one-way ANOVA; *P* = 0.07). There was also no statistically significant difference in the eGFR in premature neonates at 37 weeks PMA when compared with term infants (43.5 [39.7–48.9] vs 42.0 [38.2–50.0] mL/min/1.73 m^2^; *P* = 0.75). Table 1 shows the comparison between term control and premature neonates at 37 weeks PMA.

**Table 1:** Comparison between term (control) and premature (at 37 weeks PMA) neonates

Variable	Term (control)	Preterm (37 weeks PMA)
Number	31	53
Weight (g)	3406 (328)	2416 (328)
Gestation (weeks)	39.1 (1.2)	37.1 (0.2)
Total kidney volume (cm^3^)	26.3 (7.0)	19.2 (3.7)
Albumin:creatinine (g/mol)	5.1 [1.9–6.8]	6.7 [4.2–23.8]
Nephrin:creatinine (g/mol)	0.05 [0.03–0.14]	0.04 [0.03–0.09]
eGFR (mL/min/1.73 m^2^)	42.0 [38.2–50.0]	43.5 [39.7–48.9]

*PMA* postmenstrual age, *eGFR* estimated glomerular filtration rate

## Discussion

Premature neonates in our cohort have smaller renal volumes (hence reduced nephron number) compared to term neonates. Despite this, their eGFRs were the same as those measured in term neonates. We postulate that preterm babies, despite having fewer glomeruli, achieve a similar eGFR to their term counterparts by single nephron hyperfiltration. This sets them on a trajectory for chronic renal diseases in due course, as proposed by Brenner et al. [15].

Urinary NCR was elevated in preterm neonates in early postnatal life, but subsequently, it declined and by 37 weeks PMA, there was no significant difference between premature and term neonates in NCR. To the best of our knowledge, this is the first time urine nephrin-creatinine ratio has been measured in preterm and term neonates. Previous studies have investigated nephrin excretion in adults and in women with preeclampsia [10]. Nephrin is an integral part of the glomerular filtration barrier and any injury to podocytes results in nephrinuria. Evidence of glomerular injury as a result of prematurity has been previously demonstrated in both human and animal models using other methods [16]. Gubhaju et al. demonstrated in preterm baboons that prematurity was associated with a high prevalence of abnormal glomeruli (up to 18%) on renal histological examination. These abnormal glomeruli with a cystic Bowman’s space and shrunken glomerular tuft were present in the superficial renal cortex [17]. Similar findings have also been reported from autopsy studies conducted on deceased human preterm neonates [18] with preterm kidneys showing a greater percentage of morphologically abnormal glomeruli and a significantly larger cross-sectional area of the renal corpuscle, suggesting renal hyperfiltration.

Abnormal extra uterine glomerular development may be the result of ischaemia of the outer still developing nephrons, which are the most immature. Pappenheimer and Kinter proposed that cell-free blood is “skimmed” from the periphery of the intralobular arteries to enter the afferent arterioles of deeper glomeruli [19]. Animal studies have also demonstrated that the outermost part of the kidney receives only 20% of the blood perfusing the innermost nephrons. [20]. Thus, the most immature nephrons in the preterm kidney would be exposed to the lowest oxygen tension potentially causing ischaemic damage.

Renal toxicity from antibiotics is an additional potential cause of impaired renal function in preterm infants. In our department, a combination of beta-lactam antibiotic (penicillin) and aminoglycoside (gentamicin) is often used as first-line antibiotics for 5 to 7 days to treat early-onset sepsis. This combination is the preferred choice based on the microbial profile observed in our department [21]. The second line of preferred antibiotic, based on our microbial pattern for late-onset sepsis, is vancomycin and cephalosporin [22]. Therapeutic drug monitoring is carried out routinely on all neonates receiving aminoglycosides or vancomycin, and administration is adjusted to ensure optimal therapeutic levels. Despite this, it is possible that gentamicin and vancomycin could have contributed to glomerular injury in the early neonatal period. Patent ductus arteriosus (PDA) is a common neonatal condition in which non-steroidal anti-inflammatory drugs have been used in the past; however, we no longer use non-steroidal anti-inflammatory drugs (indomethacin and/or ibuprofen) in the management of PDA [23].

The presence of albumin in the urine could indicate either immaturity of renal glomerular function, tubular function or both. Studies designed to investigate this show that unlike their term counterparts, preterm neonates show high variability in urinary albumin levels between individuals [24]. Tsukahara et al. demonstrated that albuminuria decreased postnatally in term neonates, while it remained almost constant in preterm neonates [25]. These authors showed that glomerular permeability increased and proximal tubular protein reabsorption decreased with increasing degrees of prematurity. In another study, the same authors attempted to identify the relative contribution of tubular and glomerular dysfunction to albuminuria by measuring on days 1, 4, 7, 14, and 28 days urinary B2-microglobulin (B2M) concentration (an indicator of tubular reabsorption) in a cohort of premature neonates (gestational age 32 (2) weeks) [26]. They found that urinary B2M increased from day 1 to a peak at day 7, and then it declined. In term neonates, the B2M peaks earlier (day 4) and then falls. It is proposed that between 32 and 35 weeks of gestation, the proximal tubules mature and once this is complete, “glomerulotubular balance” is reached, and urinary albumin excretion declines. Awad et al. demonstrated that glomerular and tubular functions were also relatively impaired at birth among healthy term neonates and this corrected spontaneously by 72 h of life [24]. Urinary albumin excretion is elevated in premature neonates and shows a decline in the first 72 h of life. Normalization of glomerular function was delayed in sick premature neonates, with a slower rate of decline in urinary ACR [24]. Our findings suggest that the decline in ACR from 28 to 37 weeks represents maturation of both glomerular and tubular handling of albumin. Elevation of ACR in premature neonates at 37 week PMA when compared with term controls suggests that these babies may have residual glomerular damage. It is tempting to suggest that this is related to the higher single nephron GFR of these infants as studies in fetal and neonatal sheep have shown that hyperfiltration is associated with increased urinary protein excretion [27, 28].

The main limitation of our study is that our recruitment rate is approximately 50%. The predominant reason is that nearly half of our admissions are neonates from regional areas, who are often transferred back to health facilities nearer to home after 32 weeks PMA for ongoing care. This limits the availability of the neonates for full participation in our study. There is approximately 3 weeks in gestational age difference between term (control) and premature (at term corrected) groups. Term birth is defined at any birth after 37 weeks of gestation, up to 42 weeks. To recruit term neonates at the exact same gestation will be ideal, but more challenging in clinical practice. Furthermore, nephrogenesis is complete between 34 and 36 weeks of gestation [29]; hence, the difference in renal volume (hence nephron number) and renal function due to this disparity is likely to be minimum.

## Conclusion

Despite having smaller renal volume (and reduced nephron number), premature neonates achieve a similar eGFR as term neonates through glomerulo-hyperfiltration. They also show evidence of glomerular injury in the early neonatal period as demonstrated by nephrinuria and albuminuria. This may be due to a combination of abnormal glomerular development, ongoing ischaemia and the use of potentially nephrotoxic antibiotics. By term equivalence, nephrinuria returns to normal but albuminuria remains abnormal. Calculated glomerular filtration rate in the preterm infants is also normal at term equivalence in spite of the relatively small kidneys, strongly suggesting the presence of single nephron hyperfiltration. The hyperfiltration in preterm born infants may predispose them to continuing loss of additional nephrons and a higher risk of earlier-onset renal failure.
